# Methodological Considerations in the Pilot Study of a Novel Transcutaneous Peripheral Nerve Stimulation System for Essential Tremor

**DOI:** 10.5334/tohm.1096

**Published:** 2025-09-22

**Authors:** Muhammad Uzair

**Affiliations:** 1 Khyber Medical College, Peshawar, Pakistan

**Keywords:** Essential Tremors, Transcutaneous Peripheral Nerve Stimulation, Essential Tremor Rating Assessment Scale (TETRAS), Neuromodulation, Placebo effect, Selection Bias

**Dear editor**,

I read with great interest the pilot study by Richard Dewey III, *et al*., investigating a novel transcutaneous peripheral nerve stimulation (TPNS) system for essential tremor (ET) [1]. The reported improvements in TETRAS scores are indeed striking. The development of non-invasive neuromodulation therapies is a valuable contribution to the ET treatment landscape. However, the open-label, uncontrolled design of the study necessitates a cautious interpretation of the results, as the potential for placebo and expectation effects to influence the outcomes is considerable and merits further discussion.

First, the inclusion criteria required the participants to be proficient with smartphones and Wi-Fi. This may have created a selection bias toward a more technologically adept and potentially younger patient population. This significantly limits the generalizability of the findings to the wider ET community, which includes a substantial proportion of older individuals who may not possess these digital literacy skills yet represent a core demographic for whom new treatments are desperately needed.

Second, the intervention period was limited to 7–10 days. This duration is insufficient to evaluate the sustainability of the therapeutic effect, long-term patient adherence, or the potential for late-onset adverse events. For a chronic condition like ET and a device intended for prolonged daily use, data on efficacy and safety beyond the short-term novelty effect are crucial [2].

Finally, the primary outcome measures—TETRAS Activities of Daily Living (ADL), Performance scores, and Global Impression of Change questionnaires (PGI-I/CGI-I)—are inherently subjective. The absence of blinding in patients and investigators leaves these endpoints highly vulnerable to expectation bias. The concordance in improvement rates on the PGI-I and CGI-I (82%) is consistent with a shared positive bias rather than definitive evidence of efficacy. Although the authors acknowledge the lack of a sham control, the probable magnitude of this confound and its likely contribution to the effect size were not explored. The advanced technological nature of the intervention (“AI-powered,” “novel”) may further potentiate a placebo response.

We acknowledge that the authors have a larger, sham-controlled trial underway (NCT06235190), which will be crucial in addressing these critical methodological concerns. Until the results of that rigorous trial are available, the results of this pilot study should be viewed as a preliminary investigation of feasibility and maximum possible effect size, heavily confounded by bias, rather than a demonstration of efficacy.
